# Baseline Cerebral Ischemic Core Quantified by Different Automatic Software and Its Predictive Value for Clinical Outcome

**DOI:** 10.3389/fnins.2021.608799

**Published:** 2021-04-12

**Authors:** Zhang Shi, Jing Li, Ming Zhao, Minmin Zhang, Tiegong Wang, Luguang Chen, Qi Liu, He Wang, Jianping Lu, Xihai Zhao

**Affiliations:** ^1^Department of Radiology, Changhai Hospital, Naval Medical University, Shanghai, China; ^2^Department of Neurology, The 983^th^ Hospital of Joint Logistics Support Forces of Chinese PLA, Tianjin, China; ^3^Department of Neurology, Changhai Hospital, Naval Medical University, Shanghai, China; ^4^Institute of Science and Technology for Brain-Inspired Intelligence, Fudan University, Shanghai, China; ^5^Department of Biomedical Engineering, Center for Biomedical Imaging Research, Tsinghua University School of Medicine, Beijing, China

**Keywords:** ischemic stroke, penumbra, computed tomography, perfusion imaging, outcome, software

## Abstract

**Purpose:**

This study aims to investigate the agreement of three software packages in measuring baseline ischemic core volume (ICV) and penumbra volume (PV), and determine their predictive values for unfavorable clinical outcome in patients with endovascular thrombectomy (EVT).

**Methods:**

Patients with acute ischemic stroke who underwent computed tomographic perfusion (CTP) were recruited. Baseline CTP measurements including ICV and PV were calculated by three software packages of IntelliSpace Portal (ISP), Rapid Processing of Perfusion and Diffusion (RAPID), and fast-processing of ischemic stroke (F-STROKE). All patients received EVT, and the modified Rankin scale (mRS) at 90 days after EVT was assessed to determine the clinical outcomes (favorable: mRS = 0–2; unfavorable: mRS = 3–6). The agreement of CTP measurements among three software packages was determined using intraclass correlation coefficient (ICC). The associations between CTP measurements and unfavorable clinical outcome were analyzed using logistic regression. Receiver operating characteristic curves were conducted to calculate the area under the curve (AUC) of CTP measurements in predicting unfavorable clinical outcome.

**Results:**

Of 223 recruited patients (68.2 ± 11.3 years old; 145 males), 17.0% had unfavorable clinical outcome after EVT. Excellent agreement between F-STROKE and RAPID was found in measuring ICV (ICC 0.965; 95% CI 0.956–0.973) and PV (ICC 0.966; 95% CI 0.956–0.973). ICVs measured by three software packages were significantly associated with unfavorable clinical outcome before (odds ratios 1.012–1.018, all *P* < 0.01) and after (odds ratios 1.003–1.014, all *P* < 0.05) adjusted for confounding factors (age, gender, TOAST classification, and NIHSS on admission). In predicting unfavorable clinical outcome, ICV measured by F-STROKE showed similar performance to that measured by RAPID (AUC 0.701 vs. 0.717) but higher performance than that measured by ISP (AUC 0.629).

**Conclusions:**

The software of F-STROKE has excellent agreement with the widely used analysis tool of RAPID in measuring ICV and PV. The ischemic core volume measured by both F-STROKE and RAPID is a stronger predictor for unfavorable clinical outcome after EVT compared to ISP.

## Introduction

The current challenges in the management of acute ischemic stroke (AIS) owing to large-vessel occlusion (LVO) mainly include predicting the clinical outcome before endovascular thrombectomy (EVT), optimizing imaging analysis methods for patient selection, and evaluating the therapeutic strategy. Several recent studies demonstrated that the measurements of acute ischemic core volume (ICV) and penumbra volume (PV) have been considered as effective predictors for clinical outcome in patients receiving EVT (Bivard et al., 2015; Fiehler et al., 2019). It is well evidenced that endovascular therapy will be more beneficial for patients with a small infarct core and a large penumbra, whereas patients with a large ischemic core are at risk of developing unfavorable clinical outcome after EVT (Wannamaker et al., 2018). Therefore, accurately and reliably measuring ICV and PV is important for predicting clinical outcomes (Austein et al., 2016).

Computed tomographic perfusion (CTP) is widely used to evaluate cerebral ischemic status by calculating the perfusion maps of cerebral blood flow (CBF) and time to maximum (*T*_max_) (Austein et al., 2016). The calculation of these quantitative CTP measurements is based on different algorithms and combined in different ways to quantitatively estimate the extent of the ischemic core and penumbral tissue (Murphy et al., 2006; Wintermark et al., 2006), which have been proved to be a predictor for clinical outcomes in patients treated with reperfusion strategies (Albers et al., 2018; Nogueira et al., 2018; Vinny et al., 2018). Recently, semi-automatic commercialized CTP software, such as brain CTP package of IntelliSpace Portal (ISP), is broadly used for measuring cerebral perfusion in the routine clinical workup. Despite the widespread utility, complex postprocessing, variability in image acquisition capabilities, and a lack of standardization of perfusion thresholds have hampered the use of ISP in clinical settings (Wintermark et al., 2006). In contrast, automatic and operator-independent CTP software packages, such as Rapid Processing of Perfusion and Diffusion (RAPID), could overcome above limitations and enable routine diagnostic workup of acute stroke, (Rose et al., 2001; Yin et al., 2015; Pinto et al., 2018) which has been utilized to quantify ischemic lesions in many randomized control trials on EVT (Straka et al., 2010). However, some disadvantages of the RAPID software package still exist. Fast-processing of ischemic stroke (F-STROKE), a fully automatic processing software, which is similar to RAPID, can segment and label large vessels, parenchyma, and cerebrospinal fluid according to their time-intensity curves. F-STROKE could avoid the incorrect labeling on those low-signal volumes, including cerebrospinal fluid and arachnoid cysts. However, whether F-STROKE could improve the accuracy in measuring ICV and PV as compared with ISP and RAPID software packages is unknown.

Furthermore, investigators reported that the final infarct core and the growth of volume of infarct region were strongly associated with clinical outcomes (Austein et al., 2016; Albers et al., 2018; Nogueira et al., 2018). However, there is no consensus on the optimal CTP measurements at baseline, which could accurately predict clinical outcomes after EVT. The predictive value of ICV and PV measured by F-STROKE for clinical outcomes in patients receiving EVT is also unclear.

In the present study, we sought to investigate the agreement of F-STROKE with ISP and RAPID software packages in measuring ICV and PV using CTP data. The second aim of this study was to determine the predictive value of ICV and PV measured by F-STROKE for unfavorable clinical outcome in patients with EVT.

## Materials and Methods

### Patients

Patients with AIS who underwent CTP and referred to EVT with and without intravenous alteplase between September 2017 and December 2018 were retrospectively recruited in this study. The inclusion criteria were as follows: (1) age ≥ 18 years old; (2) AIS caused by LVO of the anterior circulation (the intracranial segment of internal carotid artery, M1 segment of the middle cerebral artery, or proximal M2 segment of the middle cerebral artery); and (3) National Institutes of Health Stroke Scale (NIHSS) ≥ 6 (acute stroke with substantial neurological deficits). Patients were excluded if they had: (1) intracranial hemorrhage; (2) the modified Rankin scale (mRS) > 2 at baseline; or (3) severe motion artifacts or poor cardiac output generating erroneous CTP data. The study protocol was approved by the Institutional Review Board of Changhai Hospital of Shanghai, and all patients provided written informed consent.

### Clinical Assessment

Clinical data including age; gender; medical history, including hypertension, hyperlipidemia, diabetes, smoking, drinking, and heart disease; and clinical scales including Glasgow Coma Scale, NIHSS, and mRS were collected from the medical record. The Trial of Org10172 in Acute Stroke Treatment (TOAST) classification was used to classify stroke types. The NIHSS assessment was performed at baseline (on admission), 1 and 24 h after EVT. The mRS was assessed at discharge and 90 days after EVT. The favorable clinical outcome after EVT was defined as a 90-days mRS of 0–2, and the unfavorable one was defined as a 90-days mRS of 3–6 (Albers et al., 2018; Nogueira et al., 2018).

### CT Examinations

All CT scans were performed on a 256-slice CT scanner (Brilliance iCT, Philips Healthcare, Cleveland, OH, United States), which included scanning parameters (non-contrast CT) and reconstruction parameters [Computed tomographic angiography (CTA) and CTP]. The scanning parameters for helical non-contrast CT were as follows: tube voltage 120 kV, tube current 350 mAs, thickness 5 mm, slice number 30, field-of-view (FOV) 250 × 250 mm^2^, and matrix 496 × 496. The imaging parameters for CTP were as follows: tube voltage 80 kV, tube current 180 mAs, whole-brain coverage in the Z-axis, FOV 220 × 220 mm^2^, matrix 512 × 512, slice thickness 5 mm, and 14 consecutive phases acquired with a temporal resolution of 4 s. A total of 50 mL of iobitridol (Xenetix-350; Guerbet, France) was intravenously injected with a flow rate of 5 mL/s, followed by a 20-mL saline flush with the same flow rate. CTA was performed after 3 min once CTP was completed with the following parameters: tube voltage 120 kV, tube current 300 mAs, FOV 220 × 220 mm^2^, matrix 512 × 512, slice thickness1 mm, and slice number 399. The same contrast agent was injected intravenously with the same flow rate to CTP, and the dose of contrast agent was 45 mL.

### Post-processing of Computed Tomographic Perfusion Images

Computed tomographic perfusion images were processed using three common commercial software independently:

#### IntelliSpace Portal

Brain CTP package of ISP Version 10.1 (Philips Health System, Best, Netherlands) offers a semi-automatic way to process CTP data, including computation of delay-insensitive parameter. ISP is a software program that requires human intervention. In this analysis, one observer (ZS) who was blinded to clinical information, and the results of the analysis using other two software utilized ISP to adjust the location of the seeds in the M1 segment of middle cerebral artery and the superior sagittal sinus to obtain the arterial inflow function, venous outflow function, and cerebral mid-line. Default 2D motion correction and brain map were used. During the calculation phase, an arrival-time-insensitive method was chosen to calculate relative CBF and *T*_max_. To make it comparable with RAPID software, the summary map was generated with impaired perfusion as *T*_max_ > 6s and severely impaired perfusion (considered as ischemic core) as relative CBF < 30% compared to the contralateral hemisphere.

#### Rapid Processing of Perfusion

Rapid processing of perfusion and diffusion (iSchemaView, Menlo Park, CA, United States) is a DICOM-In/DICOM-Out black box server, which automatically processes CT perfusion data. This software is tested and wildly used by many hospitals and research centers (Albers et al., 2018; Nogueira et al., 2018). RAPID also additionally provides a graphic user interface on the webpage to offer limited manual adjustment. It defines CBF < 30% as an ICV and relative *T*_max_ > 6s of that in normal tissue as PV, which was the inclusion criteria of the recent clinical trials of DEFUSE 3 (Albers et al., 2018) and DAWN (Nogueira et al., 2018).

#### Fast-Processing of Ischemic Stroke

Fast-processing of ischemic stroke software (Version 1.0; Neuroblem Ltd. Company, Shanghai, China) is a software running on Windows 64 server. It provides a fully automatic CTP processing combined with manual rerun mechanism in a Windows graphic user interface. It segments large vessels, parenchyma, bone, cerebrospinal fluid according to their time-intensity curves and labels the region of either ischemic core or penumbra in the time-intensity curve of parenchyma, which could avoid the incorrect labeling on those low-signal volumes, such as an arachnoid cyst. Similar to RAPID (Straka et al., 2010), F-STROKE calculates (1) *T*_max_, (2) relative cerebral blood volume (rCBV), (3) relative CBF (rCBF), and (4) mean transit time but with only arterial inflow function input.

(1) $$T_{\max}=\arg\,\max_{t}\left(r\,\left(t\right)\right)$$

(2) $$\text{rCBV}=H\,\int c_{t}\,\left(t\right)\,\text{d}\,t$$

(3) $$\mathrm{rCBF}=60\,H\,\int c_{a}\,\left(t\right)\,\text{d}\,t\,\left[\max_{t}\left(r\,\left(t\right)\right)\right]$$

(4) $$c_{t}\,\left(t\right)=c_{a}\,\left(t\right)\times r\,\left(t\right)$$

where *r*(*t*) is tissue property, *c_t_*(*t*) is the perfusion image signal, *c_a_*(*t*) is arterial inflow function, and *H* is a pre-defined constant.

To make it comparable with RAPID results, thresholds of *T*_max_ > 6s and relative CBF < 30% compared to the median relative CBF of brain tissues were configured to calculate ICV and PV, respectively. Note that using CBF with or without venous outflow function input to calculate infarct core volume is mathematically identical. Since per selection of vein output function *c*_*v*_(*t*),

$$\text{CBF}\propto \frac{\text{rCBF}}{\int c_{v}\,\left(t\right)\,\text{d}\,t}$$

And when divided by median of relative CBF, ∫*c*_*v*_(*t*)d*t* along with other constant is eliminated as a scaling factor.

### Statistical Analysis

The calculation of sample size was based on a two independent sample unpaired *t*-test with 0.80 power and 0.05 significance level (two sided). The ratio of favorable group vs. unfavorable group is 4:1. The mean and standard deviation (SD) of ICV in favorable group are 25.5 and 36.0, while the mean and SD are 64.5 and 62.9 in unfavorable group. Considering the image quality and follow-up censoring rate is 15–20% in our study, a sample size of 76 patients in favorable group and 19 in unfavorable group was needed based on PASS 15 software.

Mean values and SD were described for the continuous variables with normal distribution while median values and interquartile ranges (IQRs) were calculated for continuous variables with abnormal distribution. CTP measurements were compared among three software packages using the Friedman test with Bofferoni correction or Chi-square analysis. The agreement of CTP measurements between two software packages was determined using intraclass correlation coefficient (ICC) and Bland-Altman analysis. The agreement categories include poor (ICC < 0.4), fair to good (ICC 0.4–0.75), and excellent (ICC > 0.75) agreement. Continuous variables and categorical variables were compared between patients with unfavorable and favorable clinical outcome using independent *t* test, Mann-Whitney *U* test, or Chi-square test. Univariate and multivariate logistic regression analyses were performed to calculate the odds ratios (ORs) and corresponding 95% confidence intervals (CIs) of CTP measurements in predicting unfavorable clinical outcome. Receiver operating characteristic (ROC) curves were also conducted to calculate the area under curve (AUC) of CTP measurements in discriminating unfavorable clinical outcome. ROC curves were compared using the method developed by DeLong et al. (1988). *P* values of <0.05 were considered statistically significant. All statistical analyses were performed with IBM SPSS Statistics 24.0 (SPSS Inc an IBM Company, Chicago, IL, United States).

## Results

### Clinical Characteristics

A total of 245 patients were recruited in this study, of which 22 were excluded due to pre-stroke cases (*n* = 9), motion artifacts (*n* = 8), and poor cardiac output (*n* = 5). Of the remaining 223 patients, 145 are male, and the mean age was 68.2 ± 11.3 years old. Table 1 summarized the baseline clinical characteristics of this study population. Of 223 patients, 38 (17.0%) had unfavorable clinical outcome and 185 (83.0%) patients had favorable clinical outcome after EVT, respectively. Patients with unfavorable clinical outcome had less atherosclerotic stroke (44.7 vs. 60.0%, *P* = 0.040) and higher NIHSS [22.4 (IQR 17.0–29.0) vs. 16.5 (IQR 12.0–22.0), *P* < 0.001] at baseline compared with those with favorable clinical outcome. No significant differences were found in other clinical characteristics between patients with unfavorable and favorable clinical outcome (all *P* > 0.05).

**TABLE 1:** Baseline demographic and clinical characteristics (*n* = 232).

	Mean ± SD, median (IQR), or *n* (%)			
	All patients (*n* = 223)	Unfavorable outcome (*n* = 38)	Favorable outcome (*n* = 185)	*P* value
Age	68.2 ± 11.3	70.9 ± 10.8	67.6 ± 11.4	0.095
Sex, male	145 (65.0)	22 (57.9)	123 (66.5)	0.312
Diabetes mellitus	45 (20.2)	9 (23.7)	36 (19.5)	0.554
Hypertension	136 (61.0)	27 (71.1)	109 (58.9)	0.163
Hyperlipidemia	25 (11.2)	5 (13.2)	20 (10.8)	0.777
Smoking	42 (18.8)	5 (13.2)	37 (20.0)	0.326
Atrial fibrillation	82 (36.8)	14 (36.8)	68 (36.8)	0.992
Valvular heart disease	32 (14.3)	5 (13.2)	27 (14.6)	0.818
Coronary atherosclerotic disease	23 (10.3)	3 (7.9)	20 (10.8)	0.773
**TOAST classification**				0.040
Atherosclerotic	128 (57.4)	17 (44.7)	111 (60.0)	
Cardioembolic	67 (30)	13 (34.2)	54 (29.2)	
Other	15 (6.7)	4 (10.5)	11 (5.9)	
Undetermined	13 (5.8)	4 (10.5)	9 (4.9)	
Glasgow Coma Scale	12.0 (9.0–15.0)	11.1 (9.0–14.0)	11.6 (9.0–15.0)	0.440
NIHSS on admission	17.0 (12.0–23.0)	22.4 (17.0–29.0)	16.5 (12.0–22.0)	<0.001
**Thrombectomy strategy**				0.583
With intravenous alteplase	36 (16.1)	5 (13.2)	31 (16.8)	
Without intravenous alteplase	187 (83.9)	33 (86.8)	154 (83.2)	

*SD, standard deviation; IQR, interquartile range; TOAST, The Trial of Org10172 in Acute Stroke Treatment; NIHSS, National Institutes of Health Stroke Scale.*

During EVT treatment, 63.2% of patients with unfavorable clinical outcome were beyond 6 h, whereas this percentage in those with favorable clinical outcome was 28.1%. In contrast, during EVT treatment, the prevalence of not using intravenous alteplase in patients with unfavorable clinical outcome was lower than that of those with favorable outcome (17.6 vs. 82.4%). The NIHSS [22.3 (16.8–28.0) vs. 11.7 (IQR 7.0–17.0), *P* < 0.001] and mRS [4.9 (4.0–5.0) vs. 1.3 (IQR 0–2.0), *P* < 0.001] at discharge in patients with unfavorable clinical outcomes were significantly higher than those in patients with favorable clinical outcomes. After EVT treatment, 90.1% of patients achieved successful recanalization (mTICI 2b, *n* = 64; mTICI 3, *n* = 137), while 9.9% did not (mTICI 1, *n* = 9; mTICI 2a, *n* = 13). Additionally, there were four patients suffered from fresh hemorrhage and nine with fresh infarct after EVT treatment.

### Comparison and Agreement of Computed Tomographic Perfusion Measurements Among Three Software Packages

**FIGURE 1:**
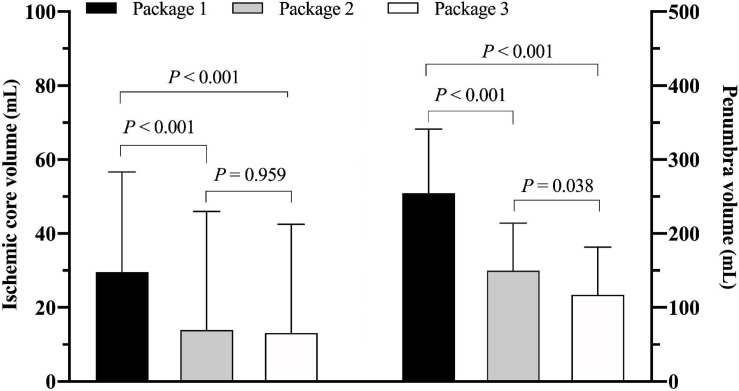
Comparison of ischemic core volume in panel **(A)** and penumbra volume in panel **(B)** among three software packages [Package 1: IntelliSpace portal (ISP); Package 2: Rapid processing of perfusion and diffusion (RAPID); Package 3: Fast-processing of ischemic stroke (F-STROKE)].

The value of ICV measured by ISP was significantly greater than thatmeasured by RAPID [29.7 mL (IQR 11.5–56.7) vs. 13.9 mL (IQR0–46.0), *P* < 0.001] and F-STROKE [29.7 mL (IQR 11.5–56.7) vs. 13.1 mL (IQR 0–42.5), *P* < 0.001] (Figure 1A). No significant difference was found in the measurement of ICV between RAPID and F-STROKE (*P* = 0.959). The PV measured by ISP was significantly greater than that measured by RAPID [254.5 mL (IQR 172.2–341.2) vs. 150.0 mL (IQR 92.0–214.0), *P* < 0.001] and F-STROKE [254.5 mL (IQR 172.2–341.2) vs. 117.1 mL (IQR 70.9–181.8), *P* < 0.001] (Figure 1B). The PV measured by F-STROKE was significantly smaller than that measured by RAPID (*P* = 0.038) (Figure 1B).

**FIGURE 2:**
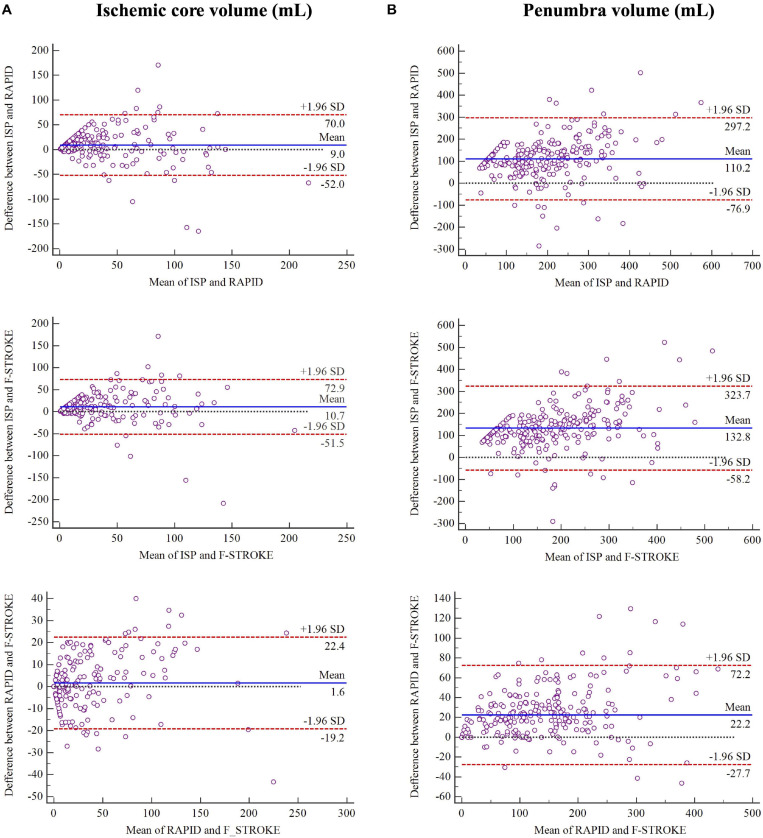
Bland-Altman plots for the agreement and bias in measuring ischemic core volume in panel **(A)** and penumbra volume in panel **(B)** between any two software packages.

In measuring ICV, the software of ISP showed fair to good agreement with RAPID (ICC 0.693; 95% CI 0.622–0.753) and F-STROKE (ICC 0.656; 95% CI 0.578–0.722). Excellent agreement was found between RAPID and F-STROKE (ICC 0.965; 95% CI 0.956–0.973) in measuring ICV. Similarly, in measuring PV, ISP showed fair to good agreement with RAPID (ICC 0.646; 95% CI 0.567–0.714) and F-STROKE (ICC 0.604; 95% CI 0.517–0.678), but excellent agreement was found between RAPID and F-STROKE (ICC 0.966; 95% CI 0.956–0.973). The Bland-Altman analysis showed that F-STROKE (mean bias 1.6; 95% CI –19.2 to 22.4) showed smaller bias than ISP (mean bias 9.0; 95% CI –52.0 to 70.0) as compared with RAPID in measuring ICV (Figure 2A). Similarly, F-STROKE (mean bias 22.2; 95% CI –27.7 to 72.2) showed smaller bias than ISP (mean bias 110.2; 95% CI –76.9 to 297.2) as compared with RAPID in measuring PV (Figure 2B).

### Association Between Computed Tomographic Perfusion Measurements and Unfavorable Clinical Outcomes

**FIGURE 3:**
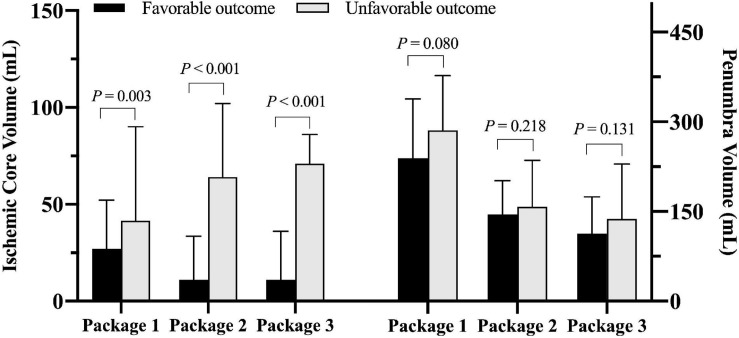
Comparison of ischemic core volume and penumbra volume measured by three software packages between patients with favorable and unfavorable clinical outcomes after endovascular thrombectomy (EVT) (Package 1: ISP; Package 2: RAPID; Package 3: F-STROKE).

Patients with unfavorable clinical outcome had significantly larger ICV measured by all three software packages compared to those with favorable clinical outcome (all *P* < 0.01, Figure 3). There was no significant difference in PV measured by any software package between patients with unfavorable and favorable clinical outcome (all *P* > 0.05, Figure 3).

Table 2 summarized the results of the regression analysis. Univariate logistic regression showed that ICV measured by any software package was significantly associated with unfavorable clinical outcome (OR 1.012 to 1.018, all *P* < 0.01). After adjusted for confounding factors of the baseline data including age, gender, TOAST classification, and NIHSS on admission, these associations remained statistically significant (OR 1.012–1.028, all *P* < 0.05). No significant associations were found between PV measured by any software package and unfavorable clinical outcome before and after adjusted for confounding factors of the baseline data (all *P* > 0.05).

**TABLE 2:** Association between CTP measurements and unfavorable clinical outcome.

	Univariate Logistic Regression			Multivariate Logistic Regression*		
	OR	95% CI	*P* value	OR	95% CI	*P* value
**Ischemic core volume**						
ISP	1.012	1.004–1.020	0.004	1.012	1.003–1.021	0.010
RAPID	1.016	1.009–1.024	<0.001	1.024	1.012–1.036	<0.001
F-STROKE	1.018	1.010–1.027	<0.001	1.028	1.014–1.042	<0.001
**Penumbra volume**						
ISP	1.002	1.000–1.015	0.083	1.001	0.996–1.004	0.975
RAPID	1.002	0.999–1.006	0.219	0.993	0.987–0.999	0.064
F-STROKE	1.003	0.999–1.007	0.133	0.992	0.986–0.999	0.058

*Adjustment: age, gender, TOAST classification, and NIHSS on administration. OR, odds ratio; CI, confidence interval.

### Receiver Operating Characteristic Analysis

**FIGURE 4:**
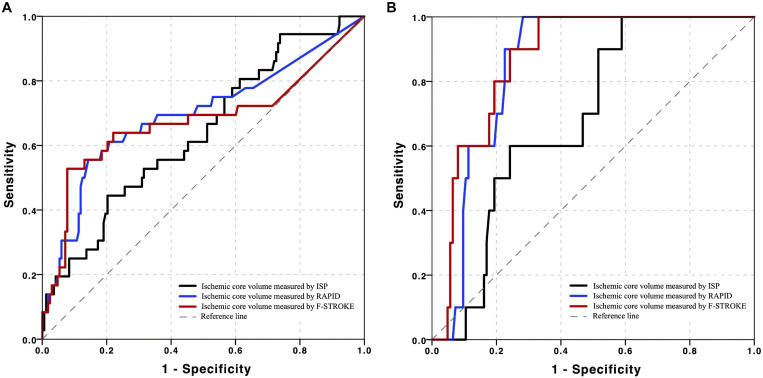
Receiver operating characteristic (ROC) curves of ischemic core volume calculated by three computed tomographic perfusion (CTP) software packages in predicting unfavorable clinical outcomes in all the patients in panel **(A)** and patients achieved complete reperfusion in panel **(B)** after EVT.

Receiver operating characteristic analysis showed that, in predicting unfavorable clinical outcome, RAPID had the highest AUC (0.717, 95% CI 0.577–0.832), followed by F-STROKE (0.701, 95% CI 0.548–0.831) and ISP (0.629, 95% CI 0.499–0.739) (Figure 4A). Furthermore, in predicting unfavorable clinical outcome among the patients with complete reperfusion after EVT (mTICI = 3), the AUC values of F-STROKE (0.879, 95% CI 0.794–0.931), RAPID (0.858, 95% 0.788–0.919), and ISP (0.717, 95% CI 0.633–0.792) were significantly improved (Figure 4B). However, there was no significant difference in the AUC values between RAPID and F-STROKE in all patients (*z* = 1.563; *P* = 0.118) or in patients achieving the complete reperfusion (*z* = 1.422; *P* = 0.155).

## Discussion

This study investigated the agreement between three software packages of ISP, RAPID, and F-STROKE in measuring ICV and PV using CTP data and determine their performance in predicting unfavorable clinical outcome after EVT. We found excellent agreement and smaller bias between F-STROKE and RAPID in measuring both ICV and PV. In predicting unfavorable clinical outcome, the ICV measured by F-STROKE showed similar strength to that measured by RAPID but higher strength than that measured by ISP. Our study indicated that the software package of F-STROKE might be an alternative analysis tool for CTP data, and the measurements of F-STROKE may have the potential in predicting adverse prognosis after EVT.

In the present study, we found that the software package of F-STROKE had an excellent agreement with RAPID in measuring ICV and PV. Similar to RAPID, F-STROKE provided a fully automatic CTP processing and defined CBF < 30% as ICV and relative *T*_max_ > 6s of that in normal tissue as PV. In the algorithm of F-STROKE, the thresholds of *T*_max_ > 6s and relative CBF < 30% compared to the median relative CBF of brain tissues were calculated with only arterial inflow function input, which is similar to RAPID. The present study also showed fair to good agreement between ISP and RAPID and F-STROKE, and the bias between F-STROKE and RAPID was smaller than that between ISP and RAPID in measuring ICV and PV. This may indicate that software package with automatic segmentation and calculation would be more accurate in measuring the volume of ischemic lesions than semi-automatic software package of ISP. Although ISP could create the CTP maps and quantify ICV and PV automatically, the regions of ischemic core and penumbra should be manually segmented by the radiologists, which may introduce subjective bias due to the experience and the measurements. Notably, our results illustrated that the PV measured by F-STROKE was significantly smaller than that measured by RAPID (*P* = 0.038). Because large vessels, parenchyma, bone, and cerebrospinal fluid were segmented by F-STROKE according to time-intensity curves and the labeled region of ischemic core in the time-intensity curve of parenchyma, this will avoid the incorrect labeling on those low-intensity volumes, such as an arachnoid cyst. Therefore, the differences identified by these two software packages could be attributed to the over the removal of cerebrospinal by RAPID (Supplementary Figure 1).

We found the baseline ICV measured by any software package was an independent predictor for unfavorable clinical outcome after EVT. Although some previous studies (Austein et al., 2016; Albers et al., 2018; Nogueira et al., 2018) showed that the final infarct core and the growth of volume of infarct region were strongly associated with the functional outcomes, the assessment of baseline ICV was still indispensable and crucial for selecting patients before undergoing either EVT or IVT and predicting their clinical outcomes (Chen et al., 2019). Recently, Panni et al. (2019) reported that increased baseline diffusion-weighted imaging (DWI) lesion volume was the strongest predictors for 90-days mortality in stroke patients with a large ICV. However, in these studies, the attention was only paid to the large ICV, and investigators did not evaluate the relationship between ICV and the subsequent clinical outcomes. In our data, patients with smaller baseline ICV would benefit from EVT compared to those with large one regardless of the penumbral size at baseline. Our finding was similar to the previous study of International Stroke Perfusion Imaging Registry (INSPIRE) (Chen et al., 2019). This indicated that ICV might have a stronger correlation with clinical outcome compared with PV. However, Chen et al. (2019) investigated that the patients with a larger baseline ICV who underwent EVT would be achieving a greater clinical benefit compared to intravenous thrombolysis alone. But our finding considered the smaller baseline ICV as a strong predictor of favorable clinical outcome in the patients suffering from EVT, which indicated the size of injured brain tissue. Furthermore, an increasing number of studies (Miles and Knuckey, 1998; Radak et al., 2017) reported that apoptosis might contribute to the death of a significant proportion of neurons during AIS, and the ischemic core of the brain experienced a sudden reduction of blood flow, overproduction of free radicals, an overload of Ca^2+^, and excitotoxicity as well as necrosis started in the first hours after the onset of AIS in the ischemic core (Radak et al., 2017). However, apoptosis within the ischemic penumbra may occur after several hours or days. Therefore, when patients suffered from EVT within the time window of the recovery (<6 h), the ischemic region of the brain, especially in the penumbral area, would be reperfused and saved, but the ischemic core would not. This may be the molecular reason why the patients who had larger ICV achieved unfavorable outcomes.

Our data showed that, in predicting unfavorable clinical outcome, the ICV measured by F-STROKE had similar performance to that measured by RAPID. These results may be explained by our findings of excellent consistency in the measurement of ICV between F-STROKE and RAPID. F-STROKE utilized the similar algorithm and standard definition of ischemic parameters with RAPID, which is a reliable CTP software validated by many RCTs (Campbell et al., 2015; Albers et al., 2018; Nogueira et al., 2018; Vinny et al., 2018). Additionally, we found that both RAPID and F-STROKE had stronger predictive value for an unfavorable clinical outcome compared to ISP. This result may be contributed to the automatic computation based on the robust algorithm of RAPID and F-STROKE. In contrast, there was subjective bias from manual segmentation of ISP. Of note, the AUC values of ICV measured by the two automatic software packages of F-STROKE and RAPID for predicting unfavorable clinical outcome in our study were higher among patients who achieved complete recanalization after EVT. Our results are in line with the previous study by Chen et al. (2019) (AUC = 0.868).

There are several limitations to our study. First, we only explored the predictive value of baseline CTP measurements by F-STROKE for clinical outcomes of acute stroke patients after EVT. Future studies are warranted to determine the performance of ICV measured by F-STROKE in predicting clinical outcome with other treatments. Second, our study only focused on the short-term clinical outcome (mRS at 90 days) after EVT. The predictive value of CTP measurements by F-STROKE for long-term clinical outcome will be explored in future studies. And the lack of golden standard for baseline ischemic core and PV might be another important limitation. Third, we didn’t do a direct comparison of the regions intensified by the three software packages since the voxels of the labeled regions could not be gotten due to the commercial reasons. And although CTP has an additional diagnostic value for detection of AIS in the anterior and posterior circulation, this study only recruited the patients with anterior circulation AIS. Further studies on both circulation ischemic status should be included together. Last, this study evaluated the consistency of two automatic software packages (F-STROKE and RAPID) in measuring CTP data. However, CTP examination has ionizing radiation and does not apply for patients with allergy to iodine or renal dysfunction. Recently, investigators demonstrated that MR perfusion, particularly dynamic susceptibility contrast (Seiler et al., 2020; Seners et al., 2020; Zhu et al., 2020) and arterial spin labeling-based perfusion techniques (Nael et al., 2013; Tan et al., 2018; Yu et al., 2018), can be reliably utilized to assess cerebral ischemic lesions. It is valuable to test the performance of F-STROKE in analyzing MR perfusion data in future studies.

## Data Availability Statement

The raw data supporting the conclusions of this article will be made available by the authors, without undue reservation.

## Ethics Statement

The studies involving human participants were reviewed and approved by Institutional Review Board of Changhai Hospital of Shanghai. The patients/participants provided their written informed consent to participate in this study.

## Author Contributions

ZS, JLi, and MZO contributed to the study concept and design, analysis and interpretation of data, drafting/revising the manuscript for content, and statistical analysis. MZN and TW were involved in analysis and interpretation of data, drafting/revising the manuscript for content, and statistical analysis. LC performed analysis and interpretation of data. QL took part in the study concept and design, analysis, and interpretation of data. HW, JLU, and XZ contributed to the study concept and design, acquisition, analysis, and interpretation of data, and drafting/revising the manuscript for content. All authors reviewed this manuscript and approved the version to be published.

## Conflict of Interest

The authors declare that the research was conducted in the absence of any commercial or financial relationships that could be construed as a potential conflict of interest.
