# Supplementary material for: Baseline Cerebral Ischemic Core Quantified by Different Automatic Software and Its Predictive Value for Clinical Outcome
Source: Front Neurosci. 2021 Apr 12;15:608799. doi: 10.3389/fnins.2021.608799 (PMC8072147; doi:10.3389/fnins.2021.608799)
Supplement: Supplementary file 1 [file Data_Sheet_1.DOCX]

**Supplementary Files**

**Title:** Baseline Cerebral Ischemic Core Quantified by Different Automatic Software and Its Predictive Value for Clinical Outcome

**Authors:** Zhang Shi, MD^1†^; Jing Li, MD^1†^; Ming Zhao, MD^2, 3†^; Minmin Zhang, MD^3^; Tiegong Wang, MD^1^; Luguang Chen, MPhil^1^; Qi Liu, MD^1^; He Wang, PhD^4*^; Jianping Lu, MD^1*^; Xihai Zhao, MD, PhD^5^

1. Department of Radiology, Changhai Hospital, Naval Medical University, Shanghai, China
2. Department of Neurology, The 983^th^ Hospital of Joint Logistics Support Forces of Chinese PLA, Tianjin, China
3. Department of Neurology, Changhai Hospital, Naval Medical University, Shanghai, China
4. Institute of Science and Technology for Brain-Inspired Intelligence, Fudan University, Shanghai, China
5. Centre for Biomedical Imaging Research, Department of Biomedical Engineering, Tsinghua University School of Medicine, Beijing, China

^†^ The three authors contributed equally to this study.

*Correspondence: Drs Jianping Lu and He Wang equally contributed to this study.

**Supplementary Figures：**

**
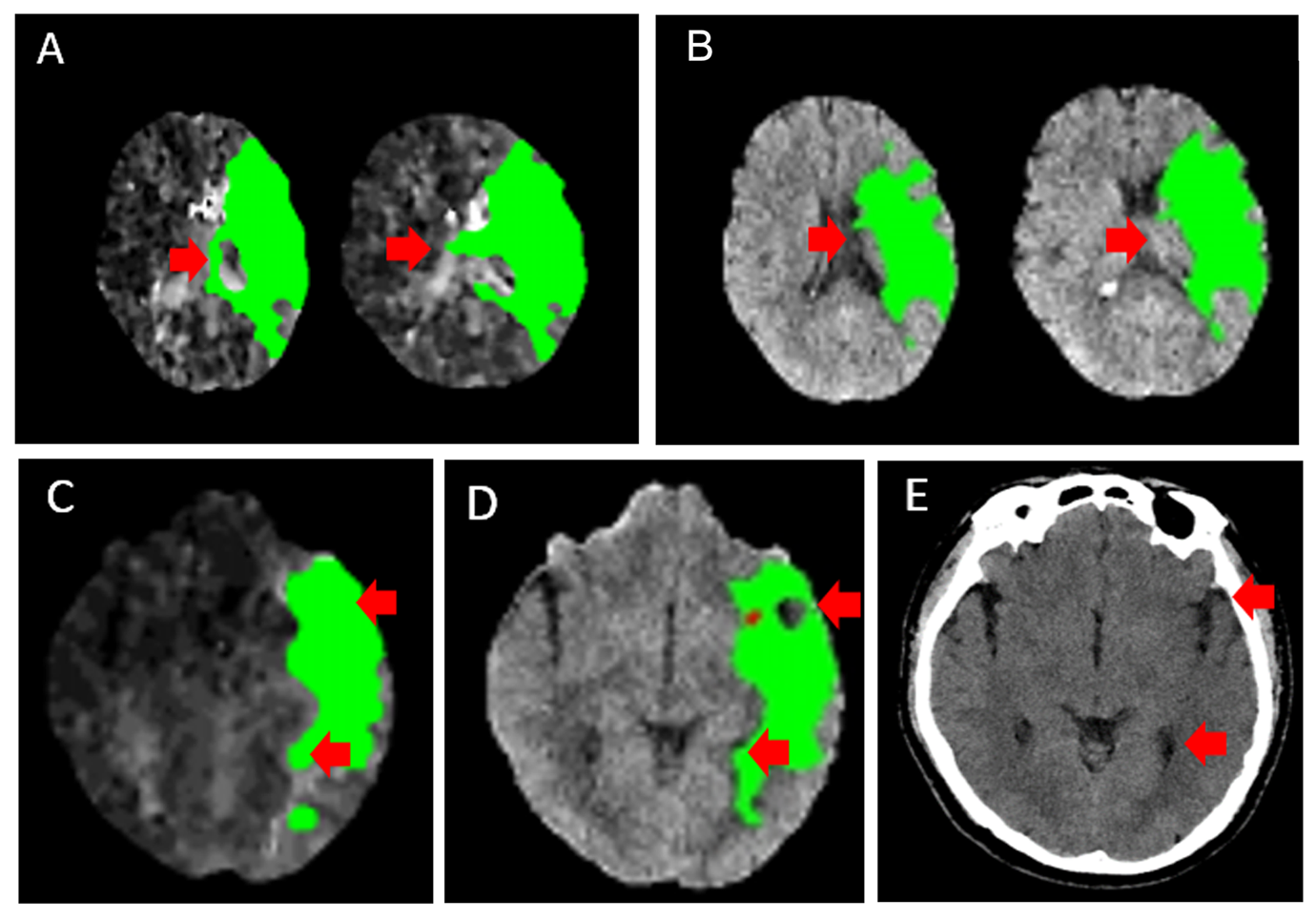
Supplementary Figure I.** The performance of removing cerebrospinal fluid of RAPID and F-STROKE are different. RAPID fails to remove some cerebrospinal fluid in lateral ventricle (A) while F-STROKE performs better on the same patient (B). For another patient, RAPID doesn’t remove the cerebrospinal fluid at lateral sulcus and lateral ventricle temporal horn (C) while F-STROKE better removes those (D). The NCCT of the same patient is shown in (E)
